# Predictors of mortality of enterococcal bacteraemia and the role of source control interventions; a retrospective cohort study

**DOI:** 10.1007/s15010-025-02561-5

**Published:** 2025-05-22

**Authors:** Virgile Zimmermann, Nicolas Fourré, Laurence Senn, Benoit Guery, Matthaios Papadimitriou-Olivgeris

**Affiliations:** 1 Infectious Diseases Service, Lausanne University Hospital, Lausanne, Switzerland; 2 Infection Prevention and Control Unit, Lausanne University Hospital, Lausanne, Switzerland; 3 Infectious Diseases Service, Hospital of Valais and Institut Central des Hôpitaux, Av. du Grand-Champsec 86, Sion, 1951 Switzerland

**Keywords:** Enterococci, Source control, Infectious diseases consultation, Sepsis, Bloodstream infection

## Abstract

**Purpose:**

To identify predictors of mortality among patients with enterococcal bacteraemia.

**Methods:**

This retrospective study was conducted at the Lausanne University Hospital, Switzerland and included adult patients with enterococcal bacteraemia from 2014 to 2023.

**Results:**

During the study period, 768 enterococcal bacteraemia episodes were included. The predominant species was *Enterococcus faecalis* (427 episodes; 56%). Sepsis or septic shock were present in 351 (46%) episodes. The overall 30-day mortality rate was 19% (148 episodes). The Cox multivariable regression model showed that age > 60 years (aHR: 1.75, 95% CI: 1.05–2.90), nosocomial infection (1.78, 1.19–2.65), sepsis or septic shock (3.67, 2.48–5.45), and not performing source control interventions within 48 h, in patients on or discussing of transitioning to limitations of care (5.91, 3.13–11.14) were associated with 30-day mortality. Conversely, infectious diseases (ID) consultation within 48 h (0.40, 0.28–0.57), appropriate antimicrobial therapy within 48 h (0.54, 0.34–0.86), and source control interventions performed within 48 h (0.22, 0.14–0.36) or not warranted (0.54; 0.34–0.86) were associated with survival. Among the 737 episodes without limitation of care, the Cox multivariable regression model showed that nosocomial infection (1.78, 1.19–2.67), sepsis or septic shock (3.76, 2.42–5.82), were associated with 30-day mortality. Conversely, ID consultation within 48 h (0.44, 0.30–0.65), appropriate antimicrobial therapy within 48 h (0.51, 0.30–0.86), and source control interventions performed within 48 h (0.25, 0.16–0.40) or not warranted (0.40; 0.26–0.61) were associated with survival.

**Conclusions:**

Our findings underscore the pivotal role of early management of enterococcal bacteraemia, including ID consultation, appropriate antimicrobial treatment initiation and performance of source control interventions.

**Supplementary Information:**

The online version contains supplementary material available at 10.1007/s15010-025-02561-5.

## Introduction

Enterococci are a common cause of bacteraemia, occurring in both community and nosocomial settings [1]. Two species are responsible for the majority of cases: *Enterococcus faecalis* and *Enterococcus faecium* [2, 3]. These species differ in their epidemiological and microbiological characteristics. *E. faecalis* is often associated with urinary tract infections and infective endocarditis, while *E. faecium* is more frequently implicated in nosocomial and abdominal infections [4, 5]. Additionally, *E. faecalis* is typically susceptible to amoxicillin, whereas *E. faecium* is often resistant to amoxicillin and more commonly linked to vancomycin resistance [4, 5]. 

The mortality rate of enterococcal bacteraemia is generally high [3–10], with worse outcomes associated with advanced age, the presence of comorbidities, sepsis or septic shock, infection caused by *E. faecium*, or resistance to ampicillin or vancomycin [3–6, 8–10]. Effective management of these infections is critical. Infectious diseases (ID) consultation and timely administration of appropriate antimicrobial therapy have been associated with improved outcomes [3, 4, 6, 8–10]. While prompt source control interventions have proven beneficial in bacteraemias caused by other pathogens [11–13], their role in enterococcal bacteraemia has been relatively underexplored.

This study aims to identify predictors of mortality in patients with enterococcal bacteraemia, focusing on the impact of early management strategies, including ID consultation, appropriate antimicrobial treatment, and timely source control interventions when indicated.

## Materials and methods

This retrospective study conducted at Lausanne University Hospital, Switzerland from January 2014 to June 2024, comprised of patients from two separate cohorts: retrospective bacteraemia cohort (January 2015 to December 2021), and the prospective cohort comprising of patients with suspected infective endocarditis (January 2022 to June 2024). The ethics committee of the Canton of Vaud approved the study (CER-VD 2021–02516, CER-VD 2017–02137).

Inclusion criteria were adult patients (≥ 18 years) and presence of at least 1 positive blood culture for *Emterococcus* spp. Exclusion criteria consisted of patients who had formally declined the use of their data, and those with incomplete medical records (including patients transferred to other hospitals at the onset of infection without follow-up data).

Blood cultures were incubated using the BacT/ALERT System (bioMerieux, Marcy l’Etoile, France). Species identification was performed using matrix-assisted laser desorption-ionization time of flight mass spectrometry (Bruker Daltonics, Bremen, Germany) from 07:00 to 19:00. Susceptibility results were obtained from the microbiology laboratory database and assessed in accordance with the European Committee on Antimicrobial Susceptibility Testing criteria [14]. 

The primary outcome of the study was the 30-day crude mortality rate. For both cohorts data on demographics (age, sex), comorbidities, severity of disease, antimicrobial treatment, source control, the presence of sepsis or septic shock, and the site of infection were manually retrieved from patients’ electronic health records by ID and internal medicine consultants.All data were reviewed by an ID consultant.

In our institution, ID consultants are informed for all positive blood cultures after species identification. In contrast to *Staphylococcus aureus* and *Candida* spp., ID consultation for enterococcal bacteraemia is not mandatory. Follow-up blood cultures until sterilization are performed in patients exhibiting persistent symptoms or suspected of having infected endocarditis. Additionally, in our institution it is a common practice for clinicians to order follow-up blood cultures for all bacteraemias involving Gram-positive cocci.

The date of collection of the first positive blood culture was defined as bacteraemia onset. A new episode was included if more than 30 days had elapsed since the cessation of antibiotic treatment for the initial bacteraemia. The classification of bacteraemia cases as community, healthcare-associated, or nosocomial followed the criteria established by Friedman et al. [15]. Chronic kidney disease was defined as an estimated glomerular filtration rate < 60 ml/min/1.73m^2^. Sepsis or septic shock were defined in accordance to the Sepsis-3 International Consensus proposition [16]. Polymicrobial bacteremia was defined as the simultaneous isolation of an additional microbial species (another *Enterococcus* spp., a non-enterococcal bacterial species, or *Candida* spp.) from at least one blood culture set collected as part of the initial blood cultures. The diagnosis of infective endocarditis was made by the Endocarditis team. The infection focus was determined by the ID consultant responsible for the case or, if no ID consultation was provided, by the treating physician based on clinical, radiological, microbiological, and surgical findings. Appropriate antimicrobial treatment was defined as the initiation of at least one antimicrobial agent with in vitro activity against the infecting isolates. Source control was considered warranted in the following situations: (1) removal of venous catheter in patients with catheter-related bacteraemia or bacteraemia of unknown origin with the presence of a venous catheter; (2) imaging-guided or surgical drainage of infected collections; (3) joint fluid drainage (arthrotomy, arthroscopy, needle aspiration); (4) cardiac surgery in patients with infective endocarditis only in the presence of heart failure indications; and (5) correction of urinary tract obstruction. Limitation of care was defined as a medical or patient-centered decision to transition from maximal care to either palliative care or a limited care approach, in which invasive interventions were withheld because they were considered disproportionate to the patient’s wishes, clinical condition, or overall prognosis.

Data analyses were performed on SPSS version 26.0 (SPSS, Chicago, IL, USA). Categorical variables were analyzed using the *chi*-square or Fisher exact test and continuous variables with Mann–Whitney *U* test. Separate analyses were performed in the entire cohort, as well as in the subgroup of episodes with warranted source control interventions and no limitations of care, and in those with *E. faecalis* or *E. faecium* bacteraemia. Univariable logistic regression models were assessed with 30-day mortality as dependent variable. Clinically relevant non collinear covariates, assessed through variance inflation factor, with a *P* < 0.1 were used in multivariable analysis. After checking Cox assumptions, a multivariable Cox proportional hazards regression models were performed with 30-day mortality as the time-to-event. To address the performance bias associated with source control interventions, we categorized patients who did not undergo source control within 48 h into two groups: those who were or discussion of transitioning to palliative care within 48 h of bacteraemia onset and those who remained on maximal care during the same period. Hazzard ratios (HRs) and 95% confidence intervals (CIs) were calculated to evaluate the strength of any association. All statistic tests were 2-tailed and *P* < 0.05 was considered statistically significant. We finally performed Kaplan-Meier curves of the survival probability of patients with enterococcal bacteraemia according to appropriate source control within 48 h from bacteraemia onset.

## Results

**Fig. 1:**
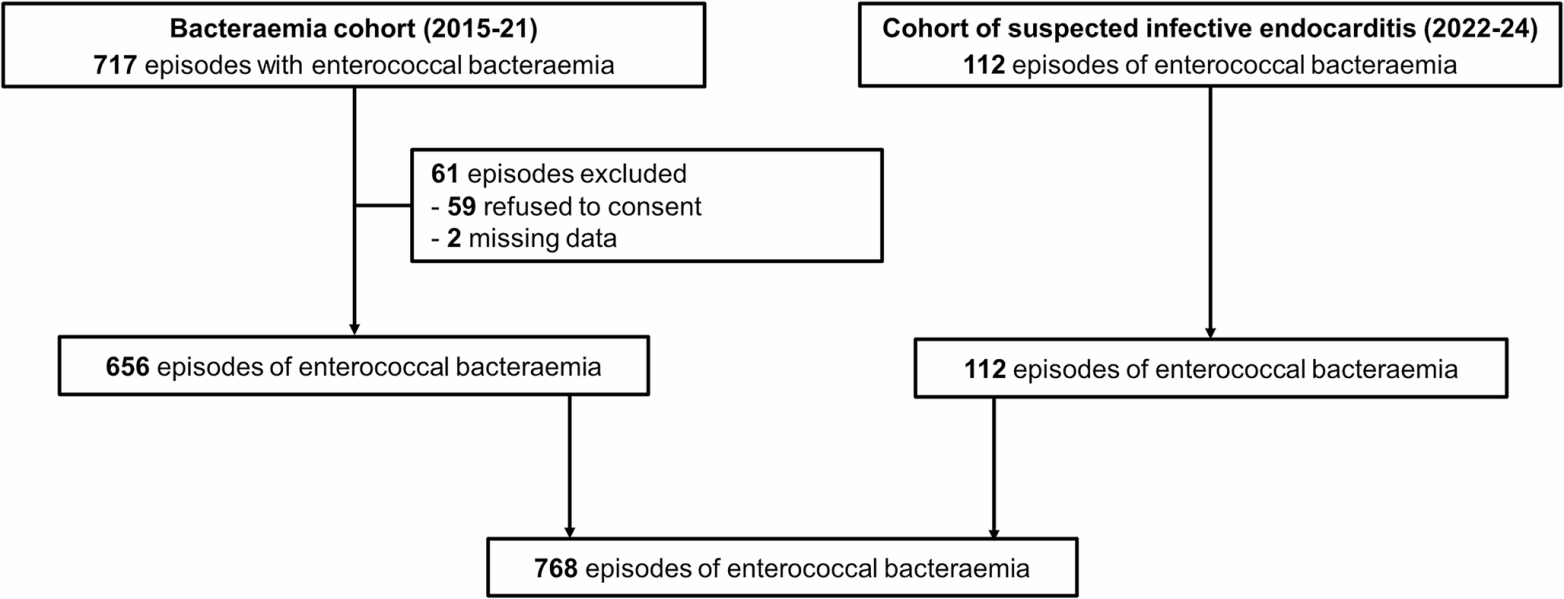
Flowchart of patients’ selection

Among 829 episodes of enterococcal bacteraemia, 768 were included (Fig. 1). The predominant species were *E. faecalis* (427 episodes; 56%), followed by *E. faecium* (321; 42%). In total, 285 (37%) isolates were resistant to ampicillin, and 31 (4%) to vancomycin. The most frequent site of infection was abdominal (310 episodes; 40%), followed by catheter-related bacteraemia (136; 18%), and infective endocarditis (115; 15%). Sepsis or septic shock were present in 351 (46%) episodes. Follow-up blood cultures were performed in 611 (81%) episodes. Persistent bacteraemia for at least 48 h was present in 71 (9%) episodes. In total, 31 episodes involved patients who were either already under palliative care or were under consideration for transitioning to limitations of care within 48 h.

The overall 30-day mortality rate was 19% (148 episodes) (Table 1). ID consultation was provided in 646 (84%) episodes, with 598/768 (78%) episodes having an ID consultation within 48 h hours of bacteraemia onset. Antimicrobial treatment was initiated within the first 48 h from bacteraemia onset in 750 (98%) episodes; in 697/768 (91%) episodes was considered appropriate. Source control interventions were warranted in 449 (60%) episodes, of which 399 (89%) episodes underwent source control, and in 270/449 (62%) episodes such procedures were performed within 48 h (Supplementary Table 1). No patient died within the first 24 h after bacteraemia onset, while 11 patients died between 24 and 48 h. Among these 11 patients, 4 had an ID consultation, all received antimicrobial treatment (7 of whom received appropriate treatment within 48 h), and 7 warranted source control interventions, none of which were performed, with 3 patients being under limitation of care.

**Table 1:** Comparison of survivors and non-survivors

	Survivors (*n* = 620)	Non-survivors (*n* = 148)	*P*
Demographics			
Male sex	446 (72)	91 (62)	0.016
Age (years)	69 (59–78)	73 (66–80)	< 0.001
Age > 60 years	442 (71)	124 (84)	0.002
Co-morbidities			
Malignancy (solid organ or haematologic)	241 (39)	69 (47)	0.093
Immunosuppression^a^	179 (29)	46 (31)	0.616
Diabetes mellitus	164 (27)	41 (28)	0.757
Chronic kidney disease (estimated glomerular filtration rate < 60 ml/min/1.73m^2^)	142 (23)	47 (32)	0.033
Obesity (body mass index ≥ 30 kg/m^2^)	115 (19)	25 (17)	0.723
Chronic obstructive pulmonary disease	72 (12)	19 (13)	0.672
Congestive heart failure	39 (6)	17 (12)	0.035
Cirrhosis	49 (8)	20 (14)	0.038
Charlson Comorbidity Index	5 (3–7)	7 (5–9)	< 0.001
Charlson Comorbidity Index > 4	358 (58)	117 (78)	< 0.001
Setting of bacteraemia onset			0.019
Community	151 (24)	21 (14)	
Healthcare-associated	110 (18)	25 (17)	
Nosocomial	359 (58)	102 (69)	
Microbiological data			
Types of enterococci			
*E. faecalis*	352 (57)	75 (51)	0.198
*E. faecium*	251 (41)	70 (47)	0.138
Non-*faecalis*, non-*faecium* enterococci	40 (7)	12 (8)	
Multiple enterococcal species	23 (4)	9 (6)	0.249
Two or more blood cultures positive (initial blood cultures)	377 (61)	79 (53)	0.113
Ampicillin-resistant	220 (36)	65 (44)	0.059
Vancomycin-resistant	26 (4)	5 (4)	0.818
Persistent bacteraemia (≥ 48 h)	58 (9)	15 (10)	0.756
Polymicrobial bacteraemia^b^	186 (30)	67 (45)	0.001
Type of infection			
Catheter-related	101 (16)	35 (24)	0.041
Urinary-tract infection	84 (14)	18 (12)	0.787
Abdominal infection	249 (40)	61 (41)	0.852
Endocarditis	101 (16)	14 (10)	0.040
Bone and joint infection	30 (5)	7 (5)	1.000
Unknown origin	37 (6)	5 (3)	0.313
Other foci	46 (7)	17 (12)	0.132
Multiple types of infection	32 (5)	9 (6)	0.684
Sepsis or septic shock	238 (38)	113 (76)	< 0.001
Septic shock	78 (13)	47 (32)	< 0.001
Management			
Infectious diseases consultation	545 (88)	101 (68)	< 0.001
Infectious diseases consultation within 48 h	512 (83)	86 (58)	< 0.001
Source control			< 0.001
Not warranted	273 (44)	46 (31)	
Warranted and performed within 48 h	242 (39)	28 (19)	
Warranted, but not performed within 48 h; no limitation of care	104 (17)	52 (35)	
Warranted, but not performed within 48 h; limitation of care^c^	1 (0.2)	22 (15)	
Antimicrobial initiation within 48 h	607 (98)	143 (97)	0.363
Appropriate antimicrobial within 48 h	578 (93)	119 (80)	< 0.001

Data are depicted as number (percentage) or median (Q1-3)

^a^ongoing immunosuppressive treatment at bacteraemia onset, intravenous chemotherapy in the 30 days prior to bacteraemia onset, AIDS, neutropenia and asplenia

^b^32 with multiple enterococcal species, 50 with streptococci, 26 with *S. aureus*, 20 with other Gram-positive bacteria, 157 with Gram-negative bacteria, 25 with *Candida* spp

^c^patients on palliative care or under discussion for palliative care

The Cox multivariable regression model (Table 2) showed that age > 60 years (aHR: 1.75, 95% CI: 1.05–2.90), nosocomial infection (1.78, 1.19–2.65), sepsis or septic shock (3.67, 2.48–5.45), and not performing source control interventions within 48 h, in patients on or discussing of transitioning to limitations of care (5.91, 3.13–11.14) were associated with 30-day mortality. Conversely, ID consultation within 48 h (0.40, 0.28–0.57), appropriate antimicrobial therapy within 48 h (0.54, 0.34–0.86), and source control interventions performed within 48 h (0.22, 0.14–0.36) or not warranted (0.54; 0.34–0.86) were associated with survival.

**Table 2:** Univariable and multivariable Cox proportional hazard regression of 30-day mortality among patients with bacteraemia due to enterococci

	Univariable analysis		Multivariable Cox regression	
	*P*	HR (95% CI)	*P*	aHR (95% CI)
Male sex	0.010	0.65 (0.47–0.90)	0.342	0.84 (0.58–1.21)
Age > 60 years	0.002	1.98 (1.28–3.06)	0.031	1.75 (1.05–2.90)
Malignancy (solid organ or haematologic)	0.092	1.32 (0.96–1.82)	0.748	1.07 (0.72–1.58)
Chronic kidney disease (estimated glomerular filtration rate < 60 ml/min/1.73m^2^)	0.026	1.48 (1.05–2.09)	0.399	1.19 (0.80–1.77)
Congestive heart failure	0.022	1.80 (1.09–2.99)	0.127	1.51 (0.89–2.56)
Cirrhosis	0.034	1.66 (1.04–2.67)	0.068	1.60 (0.97–2.65)
Charlson Comorbidity Index > 4	< 0.001	2.41 (1.63–3.57)	0.537	1.18 (0.70–1.98)
Nosocomial bacteraemia	0.014	1.45 (1.09–2.19)	0.005	1.78 (1.19–2.65)
Ampicillin-resistant	0.065	1.36 (0.98–1.88)	0.350	1.19 (0.83–1.72)
Polymicrobial bacteraemia	< 0.001	1.79 (1.29–2.47)	0.609	1.10 (0.76–1.59)
Catheter-related bacteraemia	0.031	1.52 (1.04–2.22)	0.297	1.28 (0.81–2.02)
Infective endocarditis	0.040	0.56 (0.32–0.97)	0.882	1.05 (0.55–1.99)
Sepsis or septic shock	< 0.001	4.50 (3.08–6.58)	< 0.001	3.67 (2.48–5.45)
Infectious diseases consultation within 48 h	< 0.001	0.33 (0.24–0.46)	< 0.001	0.40 (0.28–0.57)
Appropriate antimicrobial within 48 h	< 0.001	0.33 (0.22–0.50)	0.009	0.54 (0.34–0.86)
Source control				
Warranted, but not performed within 48 h; no limitation of care	reference		reference	
Warranted, but not performed within 48 h; limitation of care^a^	< 0.001	8.19 (4.90-13.69)	< 0.001	5.91 (3.13–11.14)
Warranted and performed within 48 h	< 0.001	0.27 (0.17–0.43)	< 0.001	0.22 (0.14–0.36)
Not warranted	< 0.001	0.39 (0.27–0.59)	0.009	0.54 (0.34–0.86)

aHR: adjusted hazard ratio; CI: confidence interval

^a^patients on palliative care or under discussion for palliative care

**Fig. 2:**
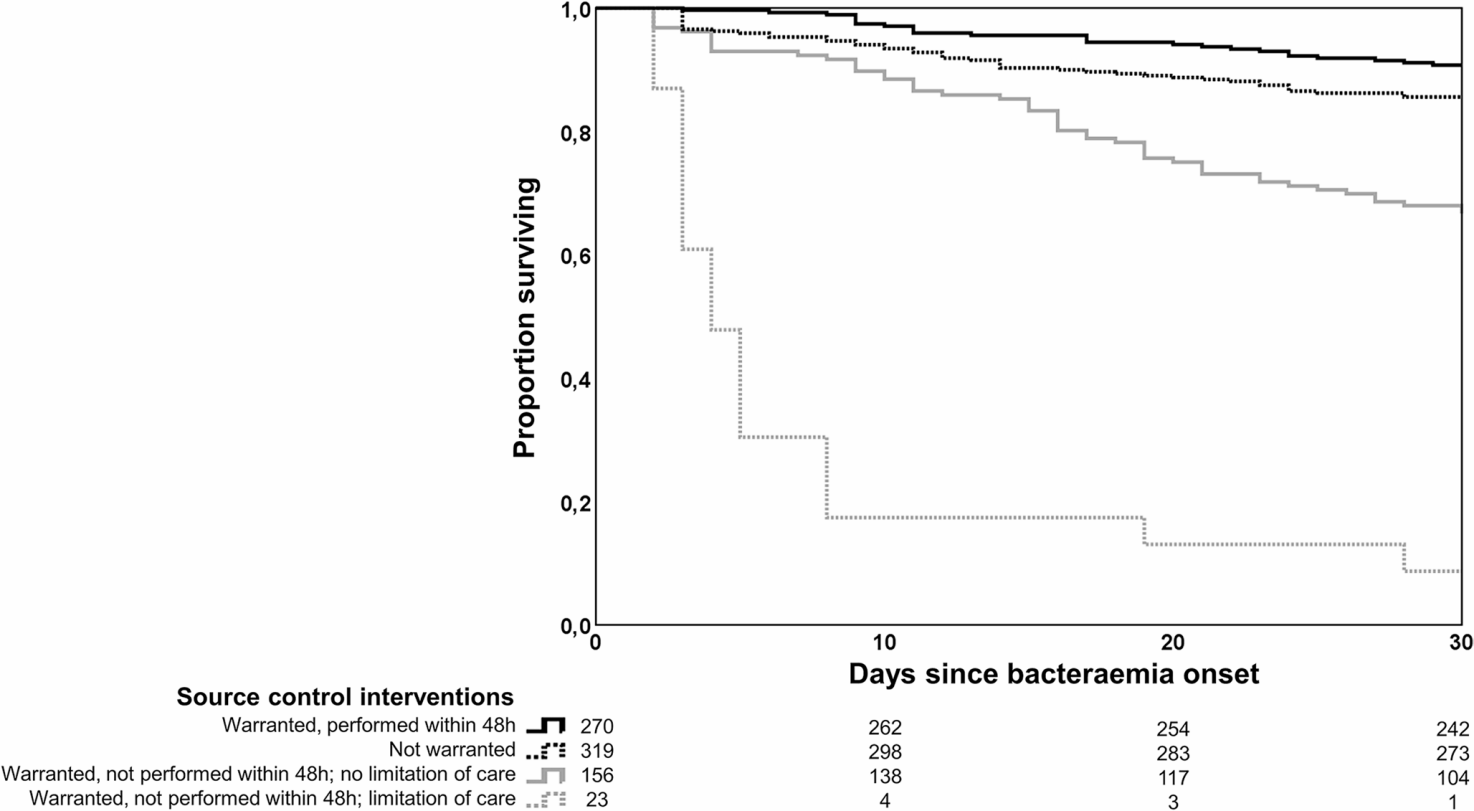
Kaplan–Meier curves of the survival probability of patients with enterococcal bacteraemia according to need and performance of early source control. Episodes that underwent source control interventions within 48 h had better outcome compared to episodes that did not, either due to limitation of care (log-rank test *P* < 0.001) or not (*P* < 0.001)

Kaplan-Meier survival curves (Fig. 2) show that episodes that underwent source control interventions within 48 h had better outcome compared to episodes that did not, either due to limitation of care (log-rank test *P* < 0.001) or not (*P* < 0.001).

Among the 737 episodes without limitation of care, 30-day mortality was 16% (118 episodes) (Table 3). The Cox multivariable regression model (Table 4) showed that nosocomial infection (aHR: 1.78, 95% CI: 1.19–2.67), sepsis or septic shock (3.76, 2.42–5.82), were associated with 30-day mortality. Conversely, ID consultation within 48 h (0.44, 0.30–0.65), appropriate antimicrobial therapy within 48 h (0.51, 0.30–0.86), and source control interventions performed within 48 h (0.25, 0.16–0.40) or not warranted (0.40; 0.26–0.61) were associated with survival.

**Table 3:** Comparison of survivors and non-survivors among 737 episodes without limitation of care

	Survivors (*n* = 619)	Non-survivors (*n* = 118)	*P*
Demographics			
Male sex	445 (72)	79 (67)	0.318
Age (years)	69 (59–78)	73 (65–81)	0.002
Age > 60 years	442 (71)	97 (82)	0.017
Co-morbidities			
Malignancy (solid organ or haematologic)	240 (39)	53 (45)	0.219
Immunosuppression^a^	178 (29)	39 (33)	0.378
Diabetes mellitus	163 (26)	37 (31)	0.261
Chronic kidney disease (estimated glomerular filtration rate < 60 ml/min/1.73m^2^)	142 (23)	39 (33)	0.026
Obesity (body mass index ≥ 30 kg/m^2^)	115 (19)	20 (17)	0.795
Chronic obstructive pulmonary disease	72 (12)	18 (15)	0.283
Congestive heart failure	39 (6)	13 (11)	0.077
Cirrhosis	49 (8)	18 (15)	0.021
Charlson Comorbidity Index	5 (3–7)	7 (5–9)	< 0.001
Charlson Comorbidity Index > 4	357 (58)	90 (76)	< 0.001
Setting of bacteraemia onset			0.025
Community	151 (24)	16 (14)	
Healthcare-associated	109 (18)	20 (17)	
Nosocomial	359 (58)	82 (70)	
Microbiological data			
Types of enterococci			
*E. faecalis*	351 (57)	58 (49)	0.157
*E. faecium*	251 (41)	56 (48)	
Non-*faecalis*, non-*faecium* enterococci	40 (7)	12 (10)	
Multiple enterococcal species	23 (4)	8 (7)	0.135
Two or more blood cultures positive (initial blood cultures)	377 (61)	63 (53)	0.151
Ampicillin-resistant	220 (36)	51 (43)	0.119
Vancomycin-resistant	26 (4)	4 (4)	1.000
Persistent bacteraemia (≥ 48 h)	58 (9)	13 (11)	0.609
Polymicrobial bacteraemia^b^	185 (30)	54 (46)	0.001
Type of infection			
Catheter-related	101 (16)	23 (20)	0.421
Urinary-tract infection	84 (14)	9 (8)	0.095
Abdominal infection	249 (40)	54 (46)	0.264
Endocarditis	101 (16)	13 (11)	0.166
Bone and joint infection	29 (5)	7 (6)	0.640
Unknown origin	37 (6)	5 (4)	0.664
Other foci	45 (7)	15 (13)	0.064
Multiple types of infection	31 (5)	8 (7)	0.499
Sepsis or septic shock	237 (38)	90 (76)	< 0.001
Septic shock	78 (13)	36 (31)	< 0.001
Management			
Infectious diseases consultation	535 (88)	89 (76)	0.002
Infectious diseases consultation within 48 h	502 (82)	74 (63)	< 0.001
Source control			< 0.001
Warranted and performed within 48 h	242 (70)	28 (35)	
Warranted, but not performed within 48 h	104 (30)	52 (65)	
Not warranted	273 (44)	38 (32)	
Antimicrobial initiation within 48 h	606 (98)	118 (100)	0.143
Appropriate antimicrobial within 48 h	578 (93)	100 (85)	0.005

Data are depicted as number (percentage) or median (Q1-3)

^a^ongoing immunosuppressive treatment at bacteraemia onset, intravenous chemotherapy in the 30 days prior to bacteraemia onset, AIDS, neutropenia and asplenia

^b^31 with multiple enterococcal species, 45 with streptococci, 22 with *S. aureus*, 20 with other Gram-positive bacteria, 149 with Gram-negative bacteria, 20 with *Candida* spp

**Table 4:** Univariable and multivariable Cox proportional hazard regression of 30-day mortality among 737 episodes without limitation of care

	Univariable analysis		Multivariable Cox regression	
	*P*	HR (95% CI)	*P*	aHR (95% CI)
Age > 60 years	0.018	1.77 (1.11–2.84)	0.142	1.52 (0.87–2.65)
Chronic kidney disease (estimated glomerular filtration rate < 60 ml/min/1.73m^2^)	0.026	1.55 (1.05–2.27)	0.680	1.09 (0.72–1.66)
Congestive heart failure	0.077	1.68 (0.95–2.99)	0.400	1.29 (0.71–2.33)
Cirrhosis	0.014	1.88 (1.14–3.11)	0.226	1.38 (0.82–2.32)
Charlson Comorbidity Index > 4	< 0.001	2.16 (1.42–3.31)	0.236	1.38 (0.82–2.32)
Nosocomial bacteraemia	0.019	1.60 (1.08–2.36)	0.005	1.78 (1.19–2.67)
Polymicrobial bacteraemia	0.001	1.86 (1.30–2.67)	0.522	1.14 (0.77–1.69)
Urinary-tract infection	0.095	0.56 (0.28–1.11)	0.243	0.68 (0.33–1.33)
Sepsis or septic shock	< 0.001	4.50 (2.95–6.88)	< 0.001	3.76 (2.42–5.82)
Infectious diseases consultation within 48 h	0.001	0.41 (0.28–0.60)	< 0.001	0.44 (0.30–0.65)
Appropriate antimicrobial within 48 h	0.001	0.43 (0.26–0.71)	0.012	0.51 (0.30–0.86)
Source control				
Warranted, but not performed within 48 h	reference		reference	
Warranted and performed within 48 h	< 0.001	0.28 (0.17–0.44)	< 0.001	0.25 (0.16–0.40)
Not warranted	< 0.001	0.33 (0.22–0.51)	< 0.001	0.40 (0.26–0.61)

aHR: adjusted hazard ratio; CI: confidence interval

Among the 427 episodes with *E. faecalis* bacteraemia and the 321 with *E. faecium* bacteraemia, 30-day mortality was 18% (75 episodes) and 22% (70 episodes), respectively (Supplementary Tables 2 and 3). The Cox multivariable regression model among the 427 episodes with *E. faecalis* bacteraemia and the 321 episodes with *E. faecium* bacteraemia appear in Supplementary Tables 4 and 5, respectively.

## Discussion

Our study investigated the factors influencing survival in patients with enterococcal bacteraemia, highlighting the critical role of early management strategies, including ID consultation, timely initiation of appropriate antimicrobial treatment, and source control interventions when warranted.

The overall 30-day mortality was 19%, which is slightly lower to that previously reported (21–28%) [3–10]. This discrepancy may be explained by several factors. In the present study, a higher proportion of episodes received appropriate antimicrobial treatment within 48 h compared to previous cohorts [4, 6, 9, 10]. Additionally, the percentage of bacteraemias caused by vancomycin-resistant enterococci (VRE) was lower in our study [5, 6, 10] and VRE cases were less likely to receive appropriate initial antimicrobial treatment in earlier studies [1, 10]. The low percentage of VRE in the present study was likely influenced by the recommendations of the Swiss Center for Infection Prevention (Swissnoso) aimed at preventing epidemic and endemic VRE spread [17]. Finally, the rate of ID consultations in our cohort was higher than what has been reported in the literature [3, 6, 10, 18]. 

Consistent with prior studies, our findings emphasize the importance of ID consultation in improving patient outcomes [3, 6, 10, 18]. ID consultations enhance overall management by facilitating earlier initiation of appropriate antimicrobial therapy and ensuring timely performance of source control interventions when needed. As shown in previous research, appropriate antimicrobial treatment was associated with improved survival [6, 8–10]. In earlier studies, inappropriate antimicrobial treatment was more common in cases of *E. faecium* bacteraemia or when the infecting isolate exhibited resistance to amoxicillin or vancomycin; [6, 9, 10] in our study, a higher proportion of episodes with *E. faecalis* bacteraemia received appropriate antimicrobial treatment within the 48-hour timeframe compared to those with *E. faecium* bacteraemia (88% *versus* 93%; *P* = 0.038). While the benefit of early source control interventions has been demonstrated in various infections, including abdominal infections, necrotizing fasciitis, sepsis, candidemia, and staphylococcal or streptococcal bacteraemia [11–13, 19–24], evidence specific to enterococcal bacteraemia is limited. Two prior small studies investigating only catheter-related enterococcal bacteraemia reported conflicting results regarding the impact of catheter removal on mortality [25, 26]. One study found an association between catheter removal and improved survival [25], while the other showed no impact [26]. However, both studies were affected by immortal-time bias, as no specific time cut-off was applied for catheter removal, and the influence of palliative care limitations on therapeutic interventions was not considered. To the best of our knowledge, the present study is the first to demonstrate improved outcomes associated with early source control across all types of enterococcal bacteraemia, and in the subgroups of *E. faecalis* and *E. faecium*. Moreover, the positive impact of timely source control procedures on outcomes remained significant even after excluding patients with limitations of care. These findings underscore the importance of a multifaceted approach to the management of enterococcal bacteraemia, which should include ID consultation, early initiation of appropriate antimicrobial therapy, and prompt implementation of source control measures.

As expected, age > 60 years was independently associated with mortality [3, 5, 6, 8]. Additionally, as previously shown, the presence of sepsis or septic shock also influenced outcome [5, 6, 9]. Factors such as age, comorbidities, and the presence of sepsis are not specific predictors of outcome in patients with enterococcal bacteraemia but are common predictors across bacteraemias caused by other species and even bacterial infections in general [11–13, 19–22, 27–29]. Furthermore, nosocomial bacteraemia was associated with higher mortality [3]. Some possible explanations are that nosocomial infections are usually caused by *E. faecium* which is more commonly amoxicillin-resistant and previously was found to be associated with higher mortality [6]. This may be due to a higher rate of early inappropriate treatment and a greater prevalence in complex abdominal infections, which, in the present study, had a low percentage of prompt source control procedures [6]. 

As previously shown, among bacteraemias caused by different Gram-positive pathogens, bacteraemia by enterococcal species was associated with high mortality, comparable to that of *Staphylococcus aureus* [1]. A possible explanation for this difference may lie in patient characteristics, such as age. In our institution, patients with enterococcal bacteraemia were older (median age of 70 years) compared to those with *S. aureus* (68 years) or streptococcal bacteraemia (66 years) [13, 30]. Furthermore, a higher percentage of enterococcal bacteraemias had a nosocomial onset (60%) compared to *S. aureus* (36%) and streptococci (32%). Moreover, enterococcal bacteraemias were associated with a higher proportion of sepsis or septic shock (46%) compared to *S. aureus* (42%) and streptococci (34%). Additionally, as previously shown [1], a lower proportion of patients with enterococcal bacteraemia received appropriate empiric antimicrobial treatment compared to those with bacteraemia caused by S. aureus or streptococci [13, 30]. Thus, bacteraemia caused by enterococcal species is more frequently associated with multiple factors linked to increased mortality, including older age, nosocomial infection, sepsis or septic shock, and inappropriate antimicrobial treatment [29]. 

Our study has several limitations. First, it is a retrospective single-center study conducted in a university institution with a low prevalence of VRE and thus not being representative of the epidemiology of non-university centers, or those with higher prevalence of vancomycin-resistant isolates. However, the study sample is large, and all data were reviewed by an ID physician. Second, the inclusion of episodes from the cohort of patients with suspected infective endocarditis may have led to an overrepresentation of cases at high risk for infective endocarditis. Third, source control interventions performance might be influenced by decisions to restrict treatment and the readiness of surgeons, or interventional radiologists to undertake such procedures, thus introducing a performance bias. However, we addressed this issue by dividing episodes that did not undergo source control procedures into two: those who were or were under discussion of transitioning to limitations of care within 48 h and those who remained on maximal care during the same period. Furthermore, we performed a separate analysis among episodes without limitations of care, which also found that performing the indicated source control procedure was associated with improved outcomes. Additionally, only 11 patients (1%) died within 48 h from bacteraemia onset, thus having a limited impact on our findings. Fourth, for cases of infective endocarditis, cardiac surgery was considered a source control intervention when performed for heart failure indications present at the onset of bacteraemia, as surgical indications related to infection control or embolism prevention are typically determined after the 48-hour threshold. Lastly, we did not stratify the analysis by different antimicrobial regimens. However, previous studies have shown that the use of vancomycin for enterococcal bacteraemia is associated with worse outcomes [31, 32]. At our institution, however, glycopeptides, linezolid, or daptomycin were used for targeted therapy only in cases of amoxicillin-resistant isolates or when beta-lactam use was contraindicated.

In conclusion, our study in patients with enterococcal bacteraemia emphasizes the importance of early ID consultation, appropriate antimicrobial treatment, and especially the prompt performance of source control interventions, when indicated. Future studies are needed to evaluate the impact of such a comprehensive approach to the enhancement of patient care and the reduction of mortality.

## Electronic supplementary material

Below is the link to the electronic supplementary material.

Supplementary Material 1

## Data Availability

The data that support the findings of this study are available from the corresponding author upon reasonable request.
