# Supplementary material for: Predictors of mortality of enterococcal bacteraemia and the role of source control interventions; a retrospective cohort study
Source: Infection. 2025 May 22;53(5):2149–58. doi: 10.1007/s15010-025-02561-5 (PMC12460546; doi:10.1007/s15010-025-02561-5)
Supplement: Supplementary file 1 — Supplementary Material 1 [file 15010_2025_2561_MOESM1_ESM.docx]

**Supplementary Table 1.** Source control procedures warranted and performed depending of infection site

| **Infection site** | **Source control warranted** | **Type of source control procedure** | **Early source control performed** |
| --- | --- | --- | --- |
| Unknown origin (n=42) | 15 (36) | Removal of central or peripheral venous catheter | 10 (67) |
| Catheter-related (n=136) | 136 (100) | Removal of central or peripheral venous catheter | 96 (71) |
| Endocarditis^a^ (n=115) | 32 (28) | Valvular replacement or removal of CIED | 22 (69) |
| Abdominal (n=310) | 179 (58) | Drainage of abscess or peritonitis, correction of biliary-tract obstruction | 107 (60) |
| Urinary-tract (n=102) | 59 (58) | Correction of urinary-tract obstruction, removal of prosthetic material | 43 (73) |
| Bone or joint (n=37) | 19 (51) | Drainage of joint fluid or abscess, drainage or replacement of osteoarticular prosthetic material | 15 (79) |
| Other (n=63) | 29 (46) | Replacement of vascular prothesis, treatment of mycotic aneurysm, drainage of empyema or abscess | 9 (31) |

Data are depicted as number (percentage)

^a^including cardiac implantable electronic device lead infection

**Supplementary Table 2.** Comparison of survivors and non-survivors among 427 episodes with *E. faecalis* bacteraemia

|  | **Survivors**  **(n=352)** | **Non-survivors**  **(n=75)** | ***P*** |
| --- | --- | --- | --- |
| Demographics |  |  |  |
| Male sex | 259 (74) | 42 (56) | 0.003 |
| Age (years) | 71 (62-79) | 76 (70-83) | <0.001 |
| Age >60 years | 275 (78) | 66 (88) | 0.057 |
| Co-morbidities |  |  |  |
| Malignancy (solid organ or haematologic) | 107 (30) | 27 (36) | 0.341 |
| Immunosuppression^a^ | 79 (22) | 20 (27) | 0.452 |
| Diabetes mellitus | 114 (32) | 24 (32) | 1.000 |
| Chronic kidney disease (estimated glomerular filtration rate <60ml/min/1.73m^2^) | 90 (26) | 28 (37) | 0.046 |
| Obesity (body mass index ≥30 kg/m^2^) | 74 (21) | 14 (19) | 0.754 |
| Chronic obstructive pulmonary disease | 39 (11) | 15 (20) | 0.053 |
| Congestive heart failure | 21 (6) | 7 (9) | 0.303 |
| Cirrhosis | 29 (8) | 5 (7) | 0.816 |
| Charlson Comorbidity Index | 5 (3-8) | 7 (5-10) | <0.001 |
| Charlson Comorbidity Index >4 | 215 (61) | 62 (83) | <0.001 |
| Setting of bacteraemia onset |  |  | 0.027 |
| Community | 114 (32) | 13 (17) |  |
| Healthcare-associated | 76 (22) | 17 (23) |  |
| Nosocomial | 162 (46) | 46 (60) |  |
| Microbiological data |  |  |  |
| Two or more blood cultures positive (initial blood cultures) | 226 (64) | 39 (52) | 0.051 |
| Ampicillin-resistant | 3 (4) | 1 (5) | 1.000 |
| Vancomycin-resistant | 0 (0) | 0 (0) | - |
| Persistent bacteraemia (≥48 hours) | 27 (8) | 9 (12) | 0.251 |
| Polymicrobial bacteraemia^b^ | 113 (32) | 41 (55) | <0.001 |
| Multiple enterococcal species | 20 (6) | 8 (11) | 0.124 |
| Type of infection |  |  |  |
| Catheter-related | 62 (18) | 21 (28) | 0.053 |
| Urinary-tract infection | 69 (20) | 12 (16) | 0.521 |
| Abdominal infection | 86 (24) | 18 (24) | 1.000 |
| Endocarditis | 88 (25) | 13 (17) | 0.179 |
| Bone and joint infection | 26 (7) | 5 (7) | 1.000 |
| Unknown origin | 18 (5) | 3 (4) | 1.000 |
| Other foci | 28 (8) | 10 (13) | 0.177 |
| Multiple types of infection | 27 (8) | 7 (9) | 0.639 |
| Sepsis or septic shock | 134 (38) | 60 (80) | <0.001 |
| Septic shock | 42 (12) | 20 (27) | 0.002 |
| Management |  |  |  |
| Infectious diseases consultation | 306 (87) | 50 (67) | <0.001 |
| Infectious diseases consultation within 48 hours | 292 (83) | 44 (59) | <0.001 |
| Source control |  |  | <0.001 |
| Not warranted | 163 (46) | 25 (33) |  |
| Warranted and performed within 48 hours | 142 (40) | 12 (16) |  |
| Warranted, but not performed within 48 hours; no limitation of care | 46 (13) | 25 (34) |  |
| Warranted, but not performed within 48 hours; limitation of care^c^ | 1 (0.3) | 13 (17) |  |
| Antimicrobial initiation within 48 hours | 347 (99) | 71 (95) | 0.055 |
| Appropriate antimicrobial within 48 hours | 338 (96) | 59 (79) | <0.001 |

Data are depicted as number (percentage) or median (Q1-3)

^a^ongoing immunosuppressive treatment at bacteraemia onset, intravenous chemotherapy in the 30 days prior to bacteraemia onset, AIDS, neutropenia and asplenia.

^b^28 with multiple enterococcal species, 38 with streptococci, 22 with *S. aureus*, 14 with other Gram-positive bacteria, 88 with Gram-negative bacteria, 9 with *Candida* spp.

^c^patients on palliative care or under discussion for palliative care

**Supplementary Table 3.** Comparison of survivors and non-survivors among 321 episodes with *E. faecium* bacteraemia

|  | **Survivors**  **(n=251)** | **Non-survivors**  **(n=70)** | ***P*** |
| --- | --- | --- | --- |
| Demographics |  |  |  |
| Male sex | 174 (69) | 46 (66) | 0.564 |
| Age (years) | 68 (58-76) | 72 (63-77) | 0.061 |
| Age >60 years | 167 (67) | 55 (79) | 0.058 |
| Co-morbidities |  |  |  |
| Malignancy (solid organ or haematologic) | 129 (51) | 42 (60) | 0.224 |
| Immunosuppression^a^ | 88 (35) | 26 (37) | 0.778 |
| Diabetes mellitus | 51 (20) | 16 (23) | 0.622 |
| Chronic kidney disease (estimated glomerular filtration rate <60ml/min/1.73m^2^) | 46 (18) | 20 (29) | 0.067 |
| Obesity (body mass index ≥30 kg/m^2^) | 37 (15) | 10 (14) | 1.000 |
| Chronic obstructive pulmonary disease | 29 (12) | 4 (6) | 0.186 |
| Congestive heart failure | 16 (6) | 9 (13) | 0.081 |
| Cirrhosis | 19 (8) | 13 (19) | 0.012 |
| Charlson Comorbidity Index | 5 (3-7) | 7 (4-9) | <0.001 |
| Charlson Comorbidity Index >4 | 142 (57) | 52 (74) | 0.008 |
| Setting of bacteraemia onset |  |  | 0.222 |
| Community | 26 (10) | 3 (4) |  |
| Healthcare-associated | 35 (14) | 8 (11) |  |
| Nosocomial | 190 (76) | 59 (84) |  |
| Microbiological data |  |  |  |
| Two or more blood cultures positive (initial blood cultures) | 142 (57) | 38 (54) | 0.786 |
| Ampicillin-resistant | 212 (85) | 65 (93) | 0.078 |
| Vancomycin-resistant | 4 (2) | 2 (3) | 0.615 |
| Persistent bacteraemia (≥48 hours) | 31 (12) | 8 (11) | 1.000 |
| Polymicrobial bacteraemia^b^ | 78 (31) | 25 (36) | 0.472 |
| Multiple enterococcal species | 19 (8) | 5 (7) | 1.000 |
| Type of infection |  |  |  |
| Catheter-related | 40 (16) | 15 (21) | 0.285 |
| Urinary-tract infection | 15 (6) | 6 (9) | 0.420 |
| Abdominal infection | 149 (59) | 42 (60) | 1.000 |
| Endocarditis | 12 (5) | 1 (1) | 0.312 |
| Bone and joint infection | 3 (1) | 0 (0) | 1.000 |
| Unknown origin | 16 (6) | 2 (3) | 0.381 |
| Other foci | 18 (7) | 6 (9) | 0.797 |
| Multiple types of infection | 4 (2) | 2 (3) | 0.615 |
| Sepsis or septic shock | 103 (41) | 49 (70) | <0.001 |
| Septic shock | 34 (14) | 36 (37) | <0.001 |
| Management |  |  |  |
| Infectious diseases consultation | 223 (89) | 51 (73) | 0.002 |
| Infectious diseases consultation within 48 hours | 206 (82) | 43 (61) | 0.001 |
| Source control |  |  | <0.001 |
| Not warranted | 97 (39) | 19 (27) |  |
| Warranted and performed within 48 hours | 99 (39) | 16 (23) |  |
| Warranted, but not performed within 48 hours; no limitation of care | 55 (22) | 25 (36) |  |
| Warranted, but not performed within 48 hours; limitation of care^c^ | 0 (0.0) | 10 (14) |  |
| Antimicrobial initiation within 48 hours | 244 (97) | 69 (99) | 0.692 |
| Appropriate antimicrobial within 48 hours | 227 (90) | 57 (81) | 0.054 |

Data are depicted as number (percentage) or median (Q1-3)

^a^ongoing immunosuppressive treatment at bacteraemia onset, intravenous chemotherapy in the 30 days prior to bacteraemia onset, AIDS, neutropenia and asplenia.

^b^24 with multiple enterococcal species, 10 with streptococci, 2 with *S. aureus*, 8 with other Gram-positive bacteria, 63 with Gram-negative bacteria, 17 with *Candida* spp.

^c^patients on palliative care or under discussion for palliative care

**Supplementary Table 4.** Univariable and multivariable Cox proportional hazard regression of 30-day mortality among 427 episodes with *E. faecalis* bacteraemia

|  | **Univariable analysis** | | **Multivariable Cox regression** | |
| --- | --- | --- | --- | --- |
|  | ***P*** | **HR (95% CI)** | ***P*** | **aHR (95% CI)** |
| Charlson Comorbidity Index >4 | 0.001 | 2.76 (1.52-5.02) | 0.128 | 1.64 (0.87-3.08) |
| Nosocomial bacteraemia | 0.033 | 1.65 (1.04-2.62) | 0.028 | 1.77 (1.06-2.94) |
| Polymicrobial bacteraemia | <0.001 | 2.33 (1.48-3.66) | 0.832 | 1.06 (0.63-1.79) |
| Sepsis or septic shock | <0.001 | 5.57 (3.16-9.82) | <0.001 | 4.34 (2.42-7.78) |
| Catheter-related bacteraemia | 0.030 | 1.75 (1.06-2.89) | 0.065 | 1.86 (0.96-3.58) |
| Infectious diseases consultation within 48 hours | 0.001 | 0.33 (0.21-0.52) | <0.001 | 0.41 (0.25-0.66) |
| Appropriate antimicrobial within 48 hours | <0.001 | 0.19 (0.11-0.34) | 0.046 | 0.48 (0.24-0.99) |
| Source control |  |  |  |  |
| Warranted, but not performed within 48 hours; no limitation of care | reference | | reference | |
| Warranted, but not performed within 48 hours; limitation of care^a^ | <0.001 | 0.19 (0.10-0.38) | <0.001 | 0.14 (0.07-0.28) |
| Warranted and performed within 48 hours | <0.001 | 8.10 (4.05-16.18) | 0.013 | 3.20 (1.28-7.96) |
| Not warranted | <0.001 | 0.27 (0.17-0.42) | 0.006 | 0.44 (0.25-0.79) |

aHR: adjusted hazard ratio; CI: confidence interval;

^a^patients on palliative care or under discussion for palliative care

**Supplementary Table 5.** Univariable and multivariable Cox proportional hazard regression of 30-day mortality among 321 episodes with *E. faecium* bacteraemia

|  | **Univariable analysis** | | **Multivariable Cox regression** | |
| --- | --- | --- | --- | --- |
|  | ***P*** | **HR (95% CI)** | ***P*** | **aHR (95% CI)** |
| Age >60 years | 0.057 | 1.74 (0.98-3.08) | 0.135 | 1.63 (0.86-3.10) |
| Charlson Comorbidity Index >4 | 0.011 | 2.01 (1.18-3.44) | 0.550 | 1.21 (0.65-2.24) |
| Ampicillin-resistant | 0.097 | 2.16 (0.87-5.36) | 0.069 | 2.38 (0.94-6.03) |
| Sepsis or septic shock | <0.001 | 3.03 (1.82-5.06) | <0.001 | 3.38 (2.00-5.72) |
| Infectious diseases consultation within 48 hours | 0.001 | 0.38 (0.24-0.62) | 0.001 | 0.44 (0.26-0.72) |
| Appropriate antimicrobial within 48 hours | 0.019 | 0.49 (0.27-0.89) | 0.035 | 0.51 (0.27-0.95) |
| Source control |  |  |  |  |
| Warranted, but not performed within 48 hours; no limitation of care | reference | | reference | |
| Warranted, but not performed within 48 hours; limitation of care^a^ | 0.004 | 0.39 (0.21-0.74) | 0.003 | 0.38 (0.20-0.73) |
| Warranted and performed within 48 hours | <0.001 | 6.77 (3.21-14.29) | <0.001 | 7.57 (3.31-17.33) |
| Not warranted | 0.020 | 0.49 (0.27-0.90) | 0.035 | 0.52 (0.28-0.95) |

aHR: adjusted hazard ratio; CI: confidence interval

^a^patients on palliative care or under discussion for palliative care
